# Supplementary material for: A lightweight YOLOv7 insulator defect detection algorithm based on DSC-SE
Source: PLoS One. 2023 Dec 20;18(12):e0289162. doi: 10.1371/journal.pone.0289162 (PMC10732421; doi:10.1371/journal.pone.0289162)
Supplement: Attachment — Submitted filename: Response to Reviewers .docx [file pone.0289162.s002.docx]

**Dear Editor and Reviewers：**

Thank you for allowing us to revise our manuscript titled 'A Lightweight YOLOv7 Insulator Defect Detection Algorithm Based on DSC-SE'. We are grateful for the constructive comments and suggestions provided by the reviewers and editors, which have greatly aided us in improving our work. We have carefully reviewed and incorporated the suggested revisions, which are highlighted in red in the revised version. We respectfully submit this updated version for your consideration. A point-to-point reply is listed below where the comments of reviewers are in blue and the replies are in black.

**Comments from the Editors:**

1. Please review your reference list to ensure that it is complete and correct. If you have cited papers that have been retracted, please include the rationale for doing so in the manuscript text, or remove these references and replace them with relevant current references. Any changes to the reference list should be mentioned in the rebuttal letter that accompanies your revised manuscript. If you need to cite a retracted article, indicate the article’s retracted status in the References list and also include a citation and full reference for the retraction notice.

**Rely:** Thank you very much for your suggestion, we have double-checked and revised the format of the references.

**Reviewer #1 Comments to Author:**

**1. Comment:** Please give descriptions of “(a), (b), (c), and (d)” in Fig. 10.

**1. Rely:** Thank you very much for your suggestion, and we have described Fig 10 in our revised paper.

Pages 25, lines 299-300: Fig 10a shows the case with low light, and Fig 10b, Fig 10c and Fig 10d show the case with background interference from towers and transmission lines.

Pages 26,lines 302-303: As seen in Fig 10c, YOLOv5s and YOLOv6 can detect small insulator defects in complex backgrounds, but with low accuracy.

**2. Comment:** Journal names in some references [3, 27, …] are missing. Please also check the spelling of author names in some references [22, 27, …]

**2. Rely:** Thanks for your valuable comments and professional suggestions. We have double-checked and revised the references in the revised paper.

**Reviewer #2 Comments to Author:**

**1 and 2. Comment:** Based on the revision, this paper is improved. However, the reference format should be re-phrased further.

**1. Rely:** We greatly appreciate your valuable suggestion. We have checked and rectified the formatting of the references in the new manuscript.

**Reviewer #3 Comments to Author:**

**1. Comment:** All my problems have been addressed well in this revised manuscript and response letter, and I have no more comments.

**1. Rely:** Thanks for your valuable comments and professional suggestions.
